# Insecticidal Triterpenes in Meliaceae III: Plant Species, Molecules, and Activities in *Munronia–Xylocarpus*

**DOI:** 10.3390/ijms25147818

**Published:** 2024-07-17

**Authors:** Meihong Lin, Xiaohui Liu, Jiaxin Chen, Jiguang Huang, Lijuan Zhou

**Affiliations:** State Key Laboratory of Green Pesticide, South China Agricultural University, Guangzhou 510642, China; linmeihonglin@163.com (M.L.); 20232023007@stu.scau.edu.cn (X.L.); 20213138141@stu.scau.edu.cn (J.C.)

**Keywords:** Meliaceae, triterpenoid molecules, insecticidal activities

## Abstract

Plants of the Meliaceae family have long attracted researchers’ interest due to their various insecticidal activities, with triterpenes being the main active ingredients. In this paper, we discuss **93** triterpenoids with insecticidal activity from **37** insecticidal plant species of **15** genera (*Munronia*, *Neobeguea*, *Pseudocedrela*, *Nymania*, *Quivisia*, *Ruagea*, *Dysoxylum*, *Soymida*, *Lansium*, *Sandoricum*, *Walsura*, *Trichilia*, *Swietenia*, *Turraea*, and *Xylocarpus*) in the family Meliaceae. Among these genera, *Trichilia* deserves further research, with twelve species possessing insecticidal activity. The **93** insecticidal molecules included **27** ring-seco limonoids (comprising **1** ring A-seco group chemical, **1** ring B-seco group chemical, **5** ring D-seco group chemicals, **14** rings A,B-seco group chemicals, **5** rings B,D-seco group chemicals, and **1** rings A,B,D-seco group chemical), **22** ring-intact limonoids (comprising **5** cedrelone-class chemicals, **6** trichilin-class chemicals, **7** havanensin-class chemicals, **2** azadirone-class chemicals, **1** vilasinin-class chemical, and **1** other chemical), **33** 2,30-linkage chemicals (comprising **25** mexicanolide-class chemicals and **8** phragmalin-class chemicals), **3** 1,n-linkage-group chemicals, **3** onoceranoid-type triterpenoids, **2** apotirucallane-type terpenoids, **2** kokosanolide-type tetranortriterpenoids, and **1** cycloartane triterpene. In particular, **59** molecules showed antifeedant activity, **30** molecules exhibited poisonous effects, and **9** molecules possessed growth regulatory activity. Particularly, khayasin, beddomei lactone, 3β,24,25-trihydroxycycloartane, humilinolides A–E and methyl-2-hydroxy-3β-isobutyroxy-1-oxomeliac-8(30)-enate showed excellent insecticidal activities, which were comparable to that of azadirachtin and thus deserved more attention. Moreover, it was noteworthy that various chemicals (such as 12α-diacetoxywalsuranolide, 11β,12α-diacetoxycedrelone, 1α,7α,12α-triacetoxy-4α-carbomethoxy-11β-hydroxy-14β,15β-epoxyhavanensin, and 11-epi-21-hydroxytoonacilide, etc.) from *Turraea* showed excellent insecticidal activity. Specially, the insecticidal activity of khayasin from *Neobeguea* against the coconut leaf beetle were similar to that of rotenone. Therefore, it was a promising candidate insecticide for the control of the coconut leaf beetle.

## 1. Introduction

The severe damage to the ecology, environment, and human health that occurs due to the usage of synthetic pesticides has necessitated a shift to natural-product-based agrochemicals that are biodegradable, eco-friendly, and safe for the environment [1]. Plants produce a diverse range of secondary metabolites, such as limonoids, alkaloids, flavonoids, and quinones, as part of their defense mechanisms against insect pests. Among these chemicals, limonoids and their precursors have attracted the attention of researchers globally because of their obvious effects on insect pests [2,3,4]. The Meliaceous plants have been proven to produce various antifeedant limonoids. Azadirachtin from the neem tree is the best example, showing strong insecticidal activities against a broad spectrum of insect species, with favorable non-toxicity toward mammalian organisms [5,6]. In general, Meliaceae, with approximately 1400 species, is a rich source of structurally diverse limonoids [3,7]. Limonoids from this family have drawn great interest among scientists due to their diverse properties [8]. Researchers have also considered these limonoids in the search for eco-friendly pesticides [9].

In our first two reviews, we discussed 218 triterpenoid molecules with insecticidal activity from 41 plant species of 13 genera (*Aglaia*, *Aphanamixis*, *Azadirachta*, *Cabralea*, *Carapa*, *Cedrela*, *Chisocheton*, *Chukrasia*, *Cipadessa*, *Entandrophragma*, *Guarea*, *Khaya*, and *Melia*) in Meliaceae [10,11]. As a continuation of these two reviews and the last part of our series of reviews of insecticidal Meliaceae species, our attention in this paper is focused on the species from 15 genera (*Munronia*, *Neobeguea*, *Pseudocedrela*, *Nymania*, *Quivisia*, *Ruagea*, *Dysoxylum*, *Soymida*, *Lansium*, *Sandoricum*, *Walsura*, *Trichilia*, *Swietenia*, *Turraea*, and *Xylocarpus*) in Meliaceae. Herein, we present a summary of the insecticidal plant species, the insecticidal molecules and their structures, the diverse insecticidal activities, the structure–activity relationship (SAR), the insecticidal mechanism of action, and the environmental toxicity of the active insecticidal molecules, to provide some meaningful hints for the exploration of these chemicals as possible lead compounds in the development of novel insecticides. 

## 2. Plant Species and Their Insecticidal Chemicals

In total, 37 insecticidal plant species (*Munronia henryi* Harms, *Neobeguea mahafalensis* J.-F. Leroy, *Pseudocedrela kotschyi* (Schweinf.) Harms, *Nymania capensis* (Thunb.) Lindb., *Quivisia papinae* Baillon ex. Grandidier, *Ruagea glabra* Triana and Planch., *Dysoxylum beddomei* Hiern, *Dysoxylum malabaricum* Bedd., *Dysoxylum hainanense* (Merr.), *Soymida febrifuga* (Roxb.) A. Juss., *Lansium domesticum* Corr., *Sandoricum koetjape* (Burm.f.) Merr., *Walsura trifoliata* (A. Juss.) Harms. (synonym: *Walsura piscidia* Roxb.), *Trichilia elegans* A. Juss., *Trichilia catigua* A.Juss., *Trichilia roka* Chiov., *Trichilia havanensis* Jacq., *Trichilia sinensis* Bentv., *Trichilia hirta* L., *Trichilia pallida* Swartz, *Trichilia claussenii* Catiguá, *Trichilia pallens* C. DC., *Trichilia emetica* Vahl., *Trichilia gilgiana* Harms, *Trichilia americana* Sessé and Moc., *Swietenia humilis* Zucc., *Swietenia macrophylla* King, *Swietenia mahogani* JACQ., *Turraea obtusifolia* Hochstetter, *Turraea abyssinica* Hochst., *Turraea floribunda* Hochstetter, *Turraea wakefieldii* Oliv., *Turraea nilotica* Kotschy and Peyr., *Turraea parvifolia* Defl., *Xylocarpus granatum* J. Koenig, *Xylocarpus moluccensis* (Lam.) M. Roem., and *Xylocarpus obovatus* (Blume) A. Juss.) from fifteen genera (*Munronia*, *Neobeguea*, *Pseudocedrela*, *Nymania*, *Quivisia*, *Ruagea*, *Dysoxylum*, *Soymida*, *Lansium*, *Sandoricum*, *Walsura*, *Trichilia*, *Swietenia*, *Turraea*, and *Xylocarpus*) are presented herein (Table 1).

### 2.1. Munronia

The genus *Munronia*, comprising 13–15 species, is naturally distributed in tropical Asia, including China, Sri Lanka, India, Indonesia, and the Philippines. Plants of this genus are dwarf shrubs or semi-shrubs. Some are used in folk medicine (for example, “Aituotuo, *Munronia henryi* Harms” in Chinese) to treat bruises, rheumatic joint pain, coughs, stomach aches, tuberculosis, and sores. Phytochemical investigations of this genus led to the discovery of ring-seco limonoids [12,13,14,15,16,17,18].

The rings A,B-seco limonoids (munroniamide and munronins B–E) isolated from *M*. *henryi* Harms showed antifeedant activity. The AR (antifeeding rate, 48 h) values of munroniamide and munronins B–E against the cabbage butterfly, *Pieris brassicae* L., at 1000 μg/mL ranged from 20.9% to 37.1%. However, the AR value of azadirachtin was 99.5%, indicating that the antifeedant activities of these compounds were lower than that of azadirachtin [19,20].

### 2.2. Neobeguea 

The genus *Neobeguea* is endemic to Madagascar. Some active extracts from plants of the genus *Neobeguea* have been reported. *Neobeguea mahafalensis* J.-F. Leroy is used as a medicinal plant in Madagascar. A decoction of the stem bark of this species is reported to treat back pain [21,22,23]. 

The andirobin-class chemical methyl angolensate and the mexicanolide-class chemical khayasin were isolated from the ethyl extract of *N. mahafalensis* seeds and exhibited marked insecticidal activity [24,25]. Methyl angolensate showed antifeedant activity against the tobacco caterpillar, *Spodoptera litura* (F.). At 1 μg/cm^2^, the PFI (percentage feeding index) value of this chemical was 65.3, while those of azadirachtin A and azadirachtin B were 27.5 and 26.7, respectively [26]. Additionally, it showed insecticidal activity at 50 mg/kg with a mortality rate of 40% against the larvae of the fall armyworm, *Spodoptera frugiperda* (J.E. Smith), indicating that it was less active than the positive control gedunin (63.3%) [27]. 

Particularly, khayasin exhibited marked insecticidal activity toward the fifth-instar larvae of the coconut leaf beetle, *Brontispa longissimi* (Gestro), with an LC_50_ value of 7.28 μg/mL at 24 h [28]. Wu et al. (2003) also confirmed the insecticidal activity of khayasin against the coconut leaf beetle at 10 μg/mL [29]. The insecticidal activity of khayasin against the coconut leaf beetle was more potent than that of azadirachtin and toosendanin, and was similar to that of rotenone. Therefore, khayasin was a promising candidate insecticide for the control of the coconut leaf beetle [28].

### 2.3. Pseudocedrela

*Pseudocedrela kotschyi* (Schweinf.) Harms is an important medicinal plant found in tropical and subtropical countries of Africa. Traditionally, *P. kotschyi* is used in the treatments of various diseases, including diabetes, malaria, abdominal pain, and diarrhea [30,31,32,33,34,35,36,37,38,39,40]. The *n*-hexane, EtOAc, and methanol extracts of *P. kotschyi* were moderately active against the tobacco whitefly, *Bemisia tabaci* (Gennadius). Moreover, the *n*-hexane and EtOAc extracts showed potent activity against the mosquito larvae [41].

The ring D-seco-type chemicals 7-deacetoxy-7-oxogedunin and 7-deacetylgedunin were isolated from the roots of *P. kotschyi* [42,43]. At 100 μg/mL, the S_50_ values (50% survival average (S_50_)/d) of 7-deacetoxy-7-oxogedunin and 7-deacetylgedunin against the leaf-cutting ant, *Atta sexdens rubropilosa* Forel, were 11 and 9 d, respectively [44].

### 2.4. Nymania

The genus *Nymania* is endemic to South Africa [45,46]. In this genus, *Nymania capensis* (Thunb.) Lindb. has been reported to be an insecticidal species [46]. Generally, it is used as a garden plant and a source of forage for goats [47]. 

The prieurianin-type chemicals prieurianin and nymania 1–4 were isolated from the bark of *N. capensis* [48]. Prieurianin inhibited the larval growth of the gram pod borer, *Helicoverpa armigera* (Hübner), and the EC_50_ value was 18.8 μg/mL [49]. Nymania-3 at the concentration of 10 μg/cm^2^ showed antifeedant activity against the jute hairy caterpillar, *Pericallia ricini* (Fab.), which was half that of azadirachtin A [50]. 

### 2.5. Quivisia

*Quivisia papinae* Baillon ex. Grandidier is an endemic Madagascan species. It is currently the sole species of the genus *Quivisia*. Azadiradione, swietenolide, and melianone have been isolated from this plant [51,52]. 

Azadiradione showed growth inhibitory activity on the tobacco budworm, *Heliothis virescens* (F.), and its EC_50_ value was 560 μg/mL [53]. At 1000 μg/mL, the 2,30-linkage-group chemical swietenolide showed antifeedant activity on the cotton leafworm, *Spodoptera littoralis* (Boisduval) [54]. Additionally, the proto-limonoid chemical melianone, at 100 μg/disc, showed a poisonous effect and antifeedant activity against the Japanese subterranean termite, *Reticulitermes speratus* (Kolbe). The mortality of the Japanese subterranean termite caused by melianone at 30 d was 95% [55].

### 2.6. Ruagea

*Ruagea* comprises 15 species in Guatemala, Costa Rica, and Panama and throughout Andean South America from Venezuela to Bolivia. All *Ruagea* species are treelets or medium-to-large trees up to 35 m high and 130 cm in dbh (diameter at breast height). The bark slash is usually pinkish and fragrant [56,57]. In this genus, *Ruagea glabra* Triana and Planch. is reported to show insecticidal activity [58].

Methyl angolensate and mexicanolide-type limonoids, including xylocarpin and ruageanins A and B, were isolated from *R. glabra* [58,59,60,61]. Xylocarpin and ruageanins A and B showed comparable antifeedant activity on *S*. *frugiperda* at 1000 μg/mL at 18 h, and the AI (antifeedant index) values were 77.8, 72.6, and 86.3, respectively. However, their antifeedant activity was lower than that of azadirachtin [58].

### 2.7. Dysoxylum

Most of the *Dysoxylum* spp. are large-sized trees with leaves containing several limonoids. *Dysoxylum beddomei* Hiern, *Dysoxylum malabaricum* Bedd., and *Dysoxylum hainanense* (Merr.) show insecticidal activities due to triterpenoids [62,63,64].

From these species, nymania-3, three rings A,B-type chemicals (dysoxylumic acids A–C), three prieurianin-type chemicals (dysoxylumins A–C), one ring A-type chemical (beddomei lactone), and one cycloartane triterpene (3β,24,25-trihydroxycycloartane) have been reported to show insecticidal activity against the cabbage butterfly, *P. rapae* [50,65,66,67,68].

Dysoxylumins A–C, dysoxylumolides A–C and dysoxylumic acid D showed antifeeding activity against the cabbage butterfly. The AR values (24 h) of dysoxylumic acids A–C at 500 μg/mL ranged from 59.4% to 78.7%, which was lower than that of azadirachtin (100%), while the AR values (24 h) of dysoxylumins A–C at 1000 μg/mL ranged from 73.8% to 77.4%, which was also lower than that of azadirachtin (100%) [65]. 

Interestingly, beddomei lactone and 3β,24,25-trihydroxycycloartane exhibited strong poisonous activity, antifeedant activity, growth inhibitory activity and oviposition deterrence activity against the rice leaf-folder, *C. medinalis*. The LC_50_ values (48 h) of the two triterpenes were 6.66 and 5.79 μg/mL, respectively, while the LC_90_ values (48 h) were 14.65 and 13.93 μg/mL, respectively [68,69]. Further studies also revealed that these two chemicals have strong larvicidal, pupicidal, and adulticidal activity against the mature and immature stages of the malarial vector *Anopheles stephensi* Liston [70]. They also affected the reproductive potential of adults by acting as oviposition deterrents against the mature and immature stages of *A. stephensi*. The highest tested concentration of both compounds (10 μg/mL) evoked more than 90% mortality and oviposition deterrence (24 h). The LC_50_ and LC_90_ values for the fourth-instar larvae, pupae, and adults of *A. stephensi* exposed to beddomei lactone and 3β,24,25-trihydroxycycloartane were less than 10 μg/mL (24 h) [62]. Therefore, beddomei lactone and 3β,24,25-trihydroxycycloartane could be used as active principles during the preparation of botanical insecticides for insect pest, like rice leaf-folder and mosquitoes.

### 2.8. Soymida

In this genus, *Soymida febrifuga* (Roxb.) A. Juss. is reported to show insecticidal activity. *S. febrifuga* is a well-known Indian medicinal plant that mainly grows in tropical areas of Asia, such as India, Malaysia, Myanmar, and southern China [71,72]. This plant has been used therapeutically for centuries in Indian traditional medicine systems for many medical purposes, including for its wound-healing properties [72,73,74].

Mexicanolide-type fissinolide and prieurianin-type swietenitin *O* were obtained from this species, and they showed antifeedant activity against the castor semilooper, *Achaea janata* Linnaeus, with AI values of 76.46 and 66.61, respectively, which were lower than that of azadirachtin (100). For *S. litura*, the AI values of these two chemicals were 61.69 and 51.93, respectively, which were also lower than that of azadirachtin (100). In addition, swietenitin *O* also showed insecticidal activity on the castor semilooper and the tobacco caterpillar, with LC_50_ values of 0.65 and 0.75 μg/cm^2^, respectively. However, the LC_50_ values of azadirachtin were 0.024 and 0.013 μg/cm^2^, respectively. Therefore, the insecticidal activity of fissinolide and swietenitin *O* was lower than that of azadirachtin [2].

### 2.9. Lansium

The duku (*Lansium domesticum* Corr.), also known as the langsat or the kokosan, is a tropical lowland fruit tree native to western Southeast Asia, from Borneo to Thailand. It occurs in the wild and in cultivated forms and is one of the most widely cultivated fruits [75,76,77,78,79]. *L. domesticum* cv *kokossan* is a higher tree commonly called “kokosan’’ in Indonesia and widely distributed in Southeast Asian countries [80]. This plant is reported to produce fruits that contain a bitter seed substance with antifeedant activity [81]. 

The methanol extract of *L. domesticum* showed strong antifeedant activity against the fourth instar larvae of the twenty-eight-spotted lady beetle, *Epilachna vigintioctopunctata* Fabricius [82]. The methanol extract of the leaves of this tree also caused the death of *A. aegypti* larvae [83].

Previous phytochemical studies on *L. domesticum* reported the presence of tetranortriterpenoids, triterpenoid glycosides, onoceranoid-type triterpenoids, and onocerandiendione-type triterpenoids [80,82,84,85,86,87]. 

The tetranortriterpenoids kokosanolide A and kokosanolide C, together with another three onoceranoid-type triterpenoids (kokosanolide B, 8,14-secogammacera-7,14-diene-3,21-dione, and 8,14-secogammacera-7,14(27)-diene-3,21-dione), were isolated from the seeds and bark of *L. domesticum*. These chemicals (except kokosanolide C) showed antifeedant activity against the fourth instar larvae of the twenty-eight-spotted lady beetle at a concentration of 1% and the antifeeding values varied from 56% to 99% [82]. Additionally, the above-mentioned insecticidal chemicals methyl angolensate and azadiradione were also isolated from *L. domesticum* [84]. The andirobin-type chemicals methyl 6-hydroxyangolensate and methyl 6-acetoxyangolensate from this plant also showed antifeedant activity against *S. littoralis* at 1000 μg/mL to some extent. The antifeeding rates were 23.8% and 25.8%, respectively [11,88,89].

### 2.10. Sandoricum

In this genus, *Sandoricum koetjape* (Burm.f.) Merr. possesses insecticidal activity [90]. *S. koetjape* is a medium-sized tree native to Southeast Asia, including Malaysia and the Philippine islands, and it bears edible fruit [91]. 

Antifeedant-activity-directed fractionation of the seed extract, with larvae of *S. frugiperda* and the European corn borer, *Ostrina nubilalis* (Hübner), resulted in the isolation of two new limonoids, sandoricin and 6-hydroxysandoricin, as the primary active constituents [92]. Sandoricin and 6-hydroxysandoricin showed antifeedant activity against European corn borer larvae at 200 μg/mL. At the same concentration, these two chemicals resulted in nearly 100% mortality before pupation. They also showed similar activity against fall armyworm larvae at 25 μg/mL [92].

### 2.11. Walsura

The genus *Walsura* comprises approximately 40 evergreen tree species widely distributed in Southeast Asia [93]. Triterpenoids and limonoids are, so far, the most abundant metabolites in this genus and have been shown to possess a wide range of biological activities, including insecticidal properties [94,95,96]. 

Among these species, *Walsura trifoliata* (A. Juss.) Harms. (synonym: *Walsura piscidia* Roxb.) is one of the most important. Recently, it has been reported to be an insecticidal plant. The bark of this plant has been commonly used in India to treat skin allergies, astringency, and diarrhea [96,97,98]. Previous chemical investigations on this plant revealed a series of tirucallane and apotirucallane triterpenoids (proto-limonoids) [96,99,100]. The apotirucallane-type terpenoids piscidinols I and L showed insecticidal activity against *A. Janata* and *S. litura*. The LC_50_ values of piscidinol I against the two insects were 40.83 and 46.55 mg/cm^2^, respectively, while those of piscidinol L were 20.00 and 22.02 mg/cm^2^, respectively. The activities of both of piscidinol I and L were quite lower than that of azadirachtin (0.024 and 0.013 mg/cm^2^, respectively) [96].

### 2.12. Trichilia

In the genus *Trichilia*, twelve species—*Trichilia elegans* A. Juss., *Trichilia catigua* A. Juss., *Trichilia roka* Chiov., *Trichilia havanensis* Jacq., *Trichilia sinensis* Bentv., *Trichilia hirta* L., *Trichilia pallida* Swartz, *Trichilia claussenii* Catiguá, *Trichilia pallens* C. DC., *Trichilia emetica* Vahl., *Trichilia gilgiana* Harms, and *Trichilia americana* Sessé and Moc.—are reported to produce insecticidal triterpenoid compounds [101,102,103,104,105,106,107,108,109,110,111]. 

The acetone extract of seeds of *T. havanensis* in solid state (resin) and its supernatant oil affected the viability and development of neonate larvae of the beet armyworm, *Spodoptera exigua* (Hübner) [101]. The aqueous extracts of leaves and twigs from *Trichilia* species (*T. casaretti, T. catigua*, *T. clausenii*, *T. elegans*, *T. pallens*, and *T. pallida*) reduced the larval weight and survival of the beet armyworm [108]. Dichloromethane extracts of the leaf and fruit of *T. pallida* showed insecticidal activity against the tomato leaf-miner, *Tuta absoluta* Meyrick [112].

In total, 18 insecticidal chemicals were isolated from this genus, including one andirobin-class (methyl angolensate), three havanensin-class limonoids (trisinlin A, 3,7-diacetylhavanensin and 1,3-diacetylhavanensin) and seven trichilin-class limonoids (trichilins A, B, C D, F, and G; and sendanin), one azadirone-class chemical (azadirone), two ring D-seco-type chemicals (photogedunin and gedunin), one ring-intact limonoid (1β,2β;21,23-diepoxy-7α-hydroxy-24,25,26,27-tetranor-apotirucalla-14,20,22-trien-3-one), and three cedrelone-class chemicals (hirtin, methyl 6,11β-dihydroxy-12α-(2-methylpropanoyloxy)-3,7-dioxo-14β,15β-epoxy-1,5-meliacadien-29-oate, and deacetylhirtin) [113,114,115]. 

Trisinlin A at 20 μg/mL showed a comparable insecticidal activity to that of azadiranchtin against the newly hatched larvae of *S. litura*, with corrected mortality rates of 96.67% (14 d). As a contrast, that of azadirachtin was 100.00% [111]. 

In a conventional leaf disk assay, trichilin A killed the third-instar larvae of the southern army worm, *Spodoptera eridania* (Stoll), over a 10-day feeding period. Trichilins B and D showed antifeedant activity against *S. eridania* at 200 and 400 μg/mL, respectively [10,114]. Additionally, trichilin B was effective on *Spodoptera exigua* Hübner, and the minimum inhibitory concentration (MIC) was 200 μg/mL [116]. Moreover, trichilins F and G showed antifeedant activity against *S. littoralis* at 300 μg/mL [113]. Another trichilin-type limonoid, sendanin, also inhibited the growth of *Pectinophora gossypiella* Saunders, *Heliothis zea* Boddie, *H. virescens*, and *S. frugiperda.* The ED_50_ values observed for a 10-day artificial diet feeding period varied from 9–60 μg/mL, with pink bollworm being the most sensitive and *H. virescens* the least [117].

The ring D-seco chemical gedunin possessed various activities toward insects. Photogedunin at 100 μg/mL was active against *A. sexdens rubropilosa* and the S_50_ value was 9 d [44]. More information can be obtained from reviews by Lin (2021) and Michel (2021), where these chemicals’ activities are summarized [10,118]. 

The azadirone-class chemical azadirone, isolated from the seeds of *T. havanensis*, showed antifeedant activity against the Colorado potato beetle, *Leptinotarsa decemlineata* Say. The AI value at 500 μg/mL (equivalent to 20.8 μg/disk) was 26.9, less than that of limonin (64 at 10 μg/disk) [119]. When treated at 1000 μg/mL for 20 h, azadirone caused a mortality rate of 30% in *L. decemlineata* [118,120]. Similarly, the ring-intact limonoid 1β,2β;21,23-diepoxy-7α-hydroxy-24,25,26,27-tetranor-apotirucalla-14,20,22-trien-3-one showed antifeedant activity against the larvae of *L. decemlineata* at 300 μg/mL [119]. 

The cedrelone-type chemical hirtin inhibited the growth of larvae of the variegated cutworm, *Peridroma saucia* (Hübner), with an EC_50_ value of 11.5 μg/mL (10 d) [121].

Additionally, the other two cedrelone-type chemicals, methyl 6,11β-dihydroxy-12α-(2-methylpropanoyloxy)-3,7-dioxo-14β,15β-epoxy-1,5-meliacadien-29-oate and deacetylhirtin, showed antifeedant activity against *H. virescens* and *H. armigera*. The feeding index values of them varied from 29 to 42 [115].

The havanensin-class compounds 3,7-diacetylhavanensin and 1,3-diacetylhavanensin showed insecticidal activity against *L. decemlineata* larvae. The mortality rate of a mixture of the two chemicals at 300 μg/mL was 50% (20 h) [118]. 

### 2.13. Swietenia

*Swietenia* contains seven known species found in the tropical and subtropical regions of the Americas and West Africa [122]. *Swietenia humilis* Zucc., *Swietenia macrophylla* King, and *Swietenia mahogani* JACQ. have been reported to show insecticidal activity [123,124,125,126].

From these species, 31 chemicals of the 2,30-linkage group (including 23 mexicanolide-class limonoids and 8 phragmalin-type limonoids), 1 ring D-seco chemical (7-deacetoxy-7-oxogedunin), and 1 rings B,D-seco group chemical (methyl 6-hydroxyangolensate) are reported to show insecticidal activity [80,88,122,125,127,128,129,130,131,132,133,134].

The 23 mexicanolide-class limonoids were swietenolide, 6-*O*-acetylswietenolide, 3,6-*O*,*O*-diacetylswietenolide, swietenine, 2-hydroxyswietenine, 2-hydroxy-6-deacetoxyswietenin, humilinolide E, humilin B, swietenin C, methyl-2-hydroxy-3β-isobutyroxy-1-oxomeliac-8(30)-enate, methyl-2-hydroxy-3β-tigloyloxy-1-oxomeliac-8(30)-enate, humilinolides A–D, swietemahonin F, 12α-acetoxy-swietephragmin C, 6-*O*-acetylswietephragmin E, 3β-*O*-detigloyl-3β-*O*-benzoyl-6-*O*-acetylswietephragmin E, 3β-*O*-detigloyl-3β-*O*-benzoyl-12α-acetoxy-swietephragmin C, swietemahonin G, 6-*O*-acetylswietemahonin G, and 6-*O*-acetyl-2-hydroxyswietenin. 

The 8 phragmalin-type limonoids were swietenialides A–E, swietephragmin I, 11-hydroxyswietephragmin B, and swietephragmin H. 

The active compounds swietenine and 2-hydroxyswietenine showed antifeedant activity against *S. frugiperda*. Their DC_50_ values (the concentrations required to produce a 50% antifeedant index) (18 h) were 2.49 and 65.8 mg/L, respectively [131]. Additionally, swietenolide, 6-*O*-acetylswietenolide, 3,6-*O*,*O*-diacetylswietenolide, and swietemahonin F showed antifeedant activity against *S. frugiperda* at 1000 μg/mL and the AI values varied from 70.2 to 94.1 [132]. Further experiments showed that the DC_50_ value of swietenolide was 80.6 mg/L at 18 h [131]. Swietenolide also showed growth inhibition activity against *H. virescens* and the tomato worm, *Manduca sexta* (Linnaeus) and the GII (growth inhibition index) values were 68.0% and 46.3% (24 h), respectively [131].

Humilinolide E, humilin B, swietenin C, methyl-2-hydroxy-3β-isobutyroxy-1-oxomeliac-8(30)-enate, and methyl-2-hydroxy-3β-tigloyloxy-1-oxomeliac-8(30)-enate were found to have insecticidal activity against the European corn borer. The survival rates to the adult stage in European corn borer treated with methyl-2-hydroxy-3β-tigloyloxy-1-oxomeliac-8(30)-enate and humilin B were more than 60% and 50%, respectively. However, those with swietenin C, humilinolide E, and methyl-2-hydroxy-3β-isobutyroxy-1-oxomeliac-8(30)-enate were less than 50%. Humilinolides E and methyl-2-hydroxy-3β-isobutyroxy-1-oxomeliac-8(30)-enate showed comparable effects to toosendandin in terms of reduction of the pupation and adult emergence [133]. Other reports also revealed that the effect of the humilinolides on reducing insect growth, increasing development time and mortality of the European corn borer, *Ostrinia nubilalis* (Hbn.) was similar to that of other limonoids. Humilinolides A–D showed insecticidal activity on the European corn borer, and the associated larval mortality rates at 50 μg/mL varied from 43.3% to 63.3%. Toosendanin at both 5 and 50 μg/mL induced only moderate larval mortalities (36% at both concentrations), but the humulinolides generally produced higher mortalities (>36% at both concentrations). Additionally, humilinolide C showed growth inhibition activity and decreased the growth of *O. nubilalis* at a concentration as low as 5 μg/mL. It also inhibited the pupation of *O. nubilalis* at 50 μg/mL, resulting in a pupation rate of only 13%. Similarly, humilinolide D inhibited the pupation of *O. nubilalis* at 50 μg/mL, resulting in a pupation rate of only 10%. Interestingly, humilinolide B delayed the individual development of male *O. nubilalis* at 50 μg/mL [123]. Therefore, humilinolides A–E and methyl-2-hydroxy-3β-isobutyroxy-1-oxomeliac-8(30)-enate showed comparable activity to toosendanin.

Swietemahonin G showed growth inhibitory activity against *Helicoverpa zea* (Boddie), *H. virescens*, and *M. sexta*, with GII values ranging from 26.1 to 37.4 [131]. Moreover, swietemahonin G and 6-*O*-acetylswietemahonin G exhibited antifeedant activity against the third-instar larvae of *S. littoralis*. They were effective at 300 and 500 μg/mL, respectively [88]. When tested in a conventional leaf disk assay against the third-instar larvae of *S. littoralis*, swietephragmin I, 2-hydroxy-6-deacetoxyswietenine, 6-*O*-acetyl-2-hydroxyswietenin, 2-hydroxyswietenine, and the andirobin-type chemical methyl 6-hydroxyangolensate showed strong activity at 500 μg/mL. The ring D-seco chemical 7-deacetoxy-7-oxogedunin and phragmalin-type limonoids (swietenialides A–E, swietephragmin H, and 11-hydroxyswietephragmin B) were found to be active at 1000 μg/mL. In total, antifeedant activities of these chemicals were weaker than those of azadirachtin [3,88,130]. 

Additionally, a mixture of 12α-acetoxy-swietephragmin C, 6-*O*-acetylswietephragmin E, 3β-*O*-detigloyl-3β-*O*-benzoyl-6-*O*-acetylswietephragmin E, and 3β-*O*-detigloyl-3β-*O*-benzoyl-12α-acetoxy-swietephragmin C, obtained from *S. macrophylla*, reduced the larval weight of *Hypsipyla grandella* (Zeller) by 53% when applied at 1.00 mg/mL [134].

### 2.14. Turraea

Plants of the *Turraea* genus are mostly trees and shrubs distributed in tropical and subtropical areas. In Africa, several *Turraea* species are used in traditional medicine to treat different ailments and are also used against insect bites [135]. Six *Turraea* species—*Turraea obtusifolia* Hochstetter, *Turraea abyssinica* Hochst., *Turraea floribunda* Hochstetter, *Turraea wakefieldii* Oliv., *Turraea nilotica* Kotschy and Peyr., and *Turraea parvifolia* Defl.—are reported to produce active insecticidal triterpenoid compounds [135,136,137,138,139,140].

From these species, two cedrelone-type chemicals (12α-diacetoxywalsuranolide and 11β,12α-diacetoxycedrelone), three 1,n-linkage-group limonoids (11β,12α-diacetoxyneotecleanin, 11β,12α-diacetoxy-14β,15β-epoxyneotecleanin, and 11β,12α-diacetoxy-1-deoxo-14β,15β-epoxy-3β-hydroxy-2-oxo-neotecleanin), four havanensin-type chemicals (1α,7α,12α-triacetoxy-4α-carbomethoxy-11β-hydroxy-14β,15β-epoxyhavanensin, 1α,7α,11β-triacetoxy-4α-carbomethoxy-12α-(2-methylpropanoyloxy)-14β,15β-epoxyhavanensin, 1α,11β-diacetoxy-4α-carbomethoxy-7α-hydroxy-12α-(2-methylpropanoyloxy)-15-oxohavanensin, and nilotin), one ring B-seco chemical (11-epi-21-hydroxytoonacilide), and one vilasinin-type chemical (1α-acetoxy-3α-propanoyloxy-vilasinin) are reported to show insecticidal activity [135,136,137].

The cedrelone-type chemicals 12α-diacetoxywalsuranolide, 11β,12α-diacetoxycedrelone, 1α,7α,12α-triacetoxy-4α-carbomethoxy-11β-hydroxy-14β,15β-epoxyhavanensin, and 11-epi-21-hydroxytoonacilide showed excellent larvicidal activities on the tomato leaf miner, *Tuta absoluta* (Meyrick). The LD_50_ values were 6.6, 5.8, 4.6, and 7.1 μg/mL (24 h), respectively. They were found to be more active compared to azadirachtin (LD_50_ value of 7.8 μg/mL) [135].

The havanensin-type chemicals 1α,7α,11β-triacetoxy-4α-carbomethoxy-12α-(2-methylpropanoyloxy)-14β,15β-epoxyhavanensin and 1α,11β-diacetoxy-4α-carbomethoxy-7α-hydroxy-12α-(2-methylpropanoyloxy)-15-oxohavanensin, and the vilasinin-type chemical 1α-acetoxy-3α-propanoyloxy-vilasinin showed larvicidal activity on the malaria mosquito, *Anopheles gambiae sensu stricto* Giles, with LD_50_ values of 4.0, 3.6 and 7.1 μg/mL (24 h), respectively, and were more potent than azadirachtin, which had an LD_50_ value of 57.1 μg/mL when tested against larvae of *A. gambiae* [136]. Additionally, nilotin showed significant antifeedant activity against the 4th instar Colorado potato beetle in no-choice feeding assays. The ED_50_ (50% feeding reduction) value was 7 μg/mL, and was comparable to that of the citrus limonoid, limonin (ED_50_ = 8 μg/mL) [137]. 

The 1,n-linkage-group limonoids 11β,12α-diacetoxyneotecleanin, 11β,12α-diacetoxy-14β,15β-epoxyneotecleanin, and 7α,12α-diacetoxy-11β-hydroxyneotecleanin showed strong dose-dependent larvicidal activity against late-third- and early-fourth-instar larvae of the mosquito *An. gambiae.* The LD_50_ (24 h) values were 7.83, 7.07, and 7.05 μg/mL, respectively [138].

### 2.15. Xylocarpus

In this genus, the three species *Xylocarpus granatum* J. Koenig, *Xylocarpus moluccensis* (Lam.) M. Roem., and *Xylocarpus obovatus* (Blume) A. Juss. are reported to produce active insecticidal triterpenoid compounds [141,142,143]. 

To date, the isolation of three ring D-seco chemicals (gedunin, photogedunin, and 7-deacetylgenudin), one ring-B-seco methyl angolensate, one rings A,B,D-type xylolactone (xyloccensin L), and three mexicanolide-type chemicals (xyloccensin P, xyloccensin Q, and khayasin) has been reported from species in this genus [3,144,145,146,147]. 

Gedunin showed insecticidal activity against *S. frugiperda*, with an LC_50_ value of 39.0 μg/mL (7 d) [148]. More information about the insecticidal activity of gedunin can be found in Lin’s review (2022) [11]. Additionally, 7-deacetylgenudin showed antifeedant activity against *Reticulitermes speratus*, with a PC_95_ (95% protective concentration) value of 113.7 μg/disc (30 d) [144].

In a conventional leaf disk assay, xyloccensins P and Q at 500 μg/mL showed potent antifeedant activity against the third instar larvae of the armyworm, *Mythimna separata* (Walker), while xyloccensins O, R, S, T, U, and V showed weak activity [149]. Xyloccensin L at 1000 μg/mL showed antifeedant activity against the cabbage butterfly, *Piece brassicae* (Linnaeus) [3]. 

Khayasin exhibited potent insecticidal activity on the coconut leaf beetle, *Brontispa longissima* Gestro, at 10 μg/mL, whereas xylomexicanolides A and B showed less activity. The lethality rates of khayasin against the coconut leaf beetle at 24 and 48 h were 75.8% and 89.1%, respectively [150]. Therefore, khayasin was a promising candidate insecticide for the control of the coconut leaf beetle [28].

## 3. Structures and Structure–Activity Relationship (SAR) of the Insecticidal Chemicals

### 3.1. Structures of the Insecticidal Chemicals

Totally, 93 insecticidal chemicals are summarized herein, including 87 tetranortriterpenoids, 3 onoceranoid-type triterpenoids, 2 apotirucallane-type protolimonoids, and 1 cycloartane. The structures of these molecules are shown in Figure 1, Figure 2, Figure 3, Figure 4, Figure 5, Figure 6, Figure 7, Figure 8, Figure 9, Figure 10, Figure 11, Figure 12, Figure 13, Figure 14, Figure 15, Figure 16, Figure 17, Figure 18, Figure 19 and Figure 20.

The 87 tetranortriterpenoids include 22 ring-intact limonoids, 27 ring-seco limonoids, 36 rearranged limonoids, and 2 kokosanolides. 

Further, the 22 ring-intact limonoids comprise 2 azadirone-class chemicals, 5 cedrelone-class chemicals, 7 havanensin-class chemicals, 6 trichilin-class chemicals, 1 vilasinin-class chemical, and 1 ring-intact limonoid (1β,2β;21,23-diepoxy-7α-hydroxy-24,25,26,27-tetranor-apotirucalla-14,20,22-trien-3-one). 

The 27 ring-seco limonoids comprise 1 ring A-seco chemical, 1 ring B-seco chemical, 5 ring D-seco chemicals, 14 rings A,B-seco chemicals, 5 rings B,D-seco chemicals, and 1 rings A,B,D-seco chemicals. 

The 36 rearranged limonoids comprise 3 chemicals of the 1,n-linkage-group and 33 chemicals of the 2,30-linkage-group. Specifically, the 33 chemicals of the 2,30-linkage-group comprise 25 mexicanolide-class chemicals and 8 phragmalin-type chemicals. 

### 3.2. Structure–Activity Relationship (SAR) of the Insecticidal Chemicals

Structure–activity relationship (SAR) or quantitative structure–activity relationship (QSAR) analysis can be used for the rational design of novel pesticides and drugs [11,113,122,151]. For the reported 93 chemicals from the 15 genera examined herein, QSAR studies of ring-intact limonoids (including those of the cedrelone class and trichilin class) and rearranged limonoids (those of the 1,n-linkage-group and the mexicanolide-class limonoids of the 2,30-linkage group) have been reported. 

It was reported that the ring-intact limonoid trichilins have potent activities, comparable to azadirachtin. A structure–activity analysis of trichilins showed that among the compounds with a 12α-OH, 12β-OH, or 12-desoxy function, compounds with 12α-OH (trichilin B) showed the most potent activity, followed by those with 12β-OH function (12β-OH compounds), while those with 12-desoxy function (trichilin D and 12a-acetoxy compounds) showed lower activity. The acetoxylation or ketonization of 7-OH or ketonization at C-12 (trichilin C) rendered the compounds inactive [10,114]. Methyl 6,11β-dihydroxy-12α-(2-methylpropanoyloxy)-3,7-dioxo-14β,15β-epoxy-1,5-meliacadien-29-oate and deacetylhirtin both have a hydroxyl moiety at C-11. For *Heliothis virescens*, the feeding index values corresponding to these chemicals were 29 and 49, respectively, while for *H. armigera*, they were 32 and 42, respectively. These results indicated that structural changes in small molecules, especially regarding the properties of the C-11 side chain, changed their antifeedant activity [115]. 

The mexicanolide-class limonoid swietenolide (belonging to the 2,30-linkage group of rearranged limonoids) showed antifeedant activity against *S. frugiperda*. However, its synthesized derivatives had better activity. The most active compounds were 3-*O*-isovalerylswietenolide and 3-*O*-isobutyrylswietenolide, with DC_50_ values of 0.19 and 0.009 mg/L, respectively. Analogs of swietenolides prepared via acylation reactions had improved antifeedant effects against *S. frugiperda* as compared with the parent compound swietenolide. A comparison of 2-hydroxyswietenine and swietenine suggested that removing the 2-OH from 2-hydroxyswietenine could improve the antifeedant activity. 3-Detigloylisoswietemahonin G, obtained via the hydrolysis and methylation of swietemahonin G, showed improved growth inhibition activity against *H. zea*, *H. virescens*, and *M. sexta* [131]. The 9,30-epoxy group (swietemahonin G) was more active than the double bond between C-9 and C-30 (2-hydroxy-6-deacetoxyswietenin, 6-O-acetyl-2-hydroxyswietenin, and 2-hydroxyswietenine) [88]. 

The 1,n-linkage-group limonoids 11β,12α-diacetoxyneotecleanin, 11β,12α-diacetoxy-14β,15β-epoxyneotecleanin, and 7α,12α-diacetoxy-11β-hydroxyneotecleanin showed strong larvicidal activity against late-third- or early-fourth-instar larvae of the mosquito *A. gambiae*. The results showed that the C-14, C-15 double bond or de-acetylation of the 11-acetate group did not alter the compounds’ larvicidal activity against the mosquitoes [138].

Additionally, comparison studies of the antifeedant activity of the tetranortriterpenoids kokosanolide A and kokosanolide C, together with three onoceranoid-type triterpenoids (kokosanolide B, 8,14-secogammacera-7,14-diene-3,21-dione, and 8,14-secogammacera-7,14(27)-diene-3,21-dione), showed that an oxygenated functional group was an important structural component for antifeedant activity. Among these five compounds, kokosanolide C lacked a ketone group and had lower antifeedant potency. However, 8,14-secogammacera-14-hydroxy-7-ene-3,21-dione had a hydroxyl group and showed the strongest activity [82]. 

## 4. Insecticidal Mechanism of Action

Plants of these fifteen genera in family Meliaceae contains insect growth regulators and antifeedants against various insect pests. However, studies on the insecticidal mechanism of action (MOA) of triterpenoids from these fifteen genera are still scarce. Relevant studies in the literature have mainly focused on the MOAs of beddomei lactone, azadirone, 3β,24,25-trihydroxycycloartane, 3,7-diacetylhavanensin, and 1,3-diacetylhavanensin. 

Presently, it is known that beddomei lactone and 3β,24,25-trihydroxycycloartane inhibit gut enzymes including the acid phosphatase, alkaline phosphatase, and adenosine triphosphatase of the rice leaf-folder. Further work was needed to elucidate the effect of these triterpenoids on midgut enzymes, especially midgut alkaline phosphatase and acid phosphatase, as they are the primary hydrolytic enzymes found in the gut of many lepidopteran insects.

Meanwhile, it was reported that a mixture of 3,7-diacetylhavanensin and 1,3-diacetylhavanensin applied at 300 μg/mL caused a reduction in protease and esterase activities during the treatment period. The activities of glutathione *S*-transferase and poly-substrate mono-oxygenases significantly increased in the treatment. However, azadirone decreased esterase activity and increased glutathione *S*-transferase activity during the treatment period when applied at 1000 μg/mL, whereas protease and poly-substrate mono-oxygenases activities were not affected [118,120]. 

Generally, the majority of triterpenoids tested showed antifeedant activity to some extent. The triterpenoids affected the digestion and absorption of ingested food. These responses may also be explained because of the disruption in the neuroendocrine center of molting in insects [70]. Until now, it has been clear that triterpenoids have different MOA depending on the test insect species and that they can exhibit both antifeedant and toxic modes of action, e.g., azadirachtin, which could reduce relative growth rate, relative consumption rate, digestibility, and efficiency of conversion of digested food, and so on. It can also act as a chronic toxin. Therefore, the MOA of these triterpenoids is quite complicated and thus more research should be conducted on the MOA of these triterpenoids.

## 5. Environmental Toxicity

In practice, various extracts from plants in Meliaceae have been used as traditional medicines. The ethno-medical uses of the plants are as varied as the different cultures and geographical locations of the people who use them. For example, *T. emetica*, a plant native to Africa, is used in traditional medicine to treat various ailments, such as abdominal pains, dermatitis, hemorrhoids, jaundice, and chest pain. This species is also known as Natal Mahogany and is used for its emetic, diuretic, and purgative properties and for the induction of labor [152,153]. Many plants in *Dysoxylum* are traditional medicines in Fiji, Papua New Guinea, and New Zealand for the treatment of fever, spasm, facial deformation, and limb numbness [154,155]. In addition to medicinal uses, the plants are also used in horticulture (for ornamental purposes and shade), as food, and for making wooden items and implements. 

Generally, these extracts or chemicals are comparatively safe for the environment, human beings, and entomophagous predators. 

There are some references in the literature concerning the environmental toxicity of extracts or certain isolated chemicals from these 15 genera. 

For example, a crude aqueous extract from *T*. *emetica* root did not show toxicity (LC_50_ > 1000 μg/mL) in a brine shrimp bioassay. Trichilins from *T. emetica* have attracted much attention for their various bioactivities including insect growth regulatory, antifeedant, bactericidal, antifungal and antiviral activity. The aqueous extract by decoction of root of *T. emetica* has been used as a traditional drug for respiratory infections. It is noteworthy that this fraction was effective at the same concentration as ampicillin against some strains of *Staphylococcus aureus*. In the rat in vivo model, the treatment with *T. emetica* extracts was effective in protecting against CCl4-induced liver damage [156].

A low cytotoxicity was observed for the humilinolides against three human cell lines. Particularly, the cytotoxic activity of humilinolides A-D was determined against three human solid tumor cell lines, lung carcinoma (A-549), breast carcinoma (MCF-7), and colon adenocarcinoma (HT-29). They showed low but measurable cytotoxic effects at concentrations several orders of magnitude higher than Adriamycin [123].

In a brine shrimp assay, extracts from the bark and leaves (1 mg/mL) of *N. capensis*, *T. floribunda*, and *T. obtusifolia* demonstrated minimal to no toxicity. *T. floribunda* was found to have the least effect on the brine shrimp with zero percentage mortality recorded at both 24 and 48 h. All percentage mortality rates observed were below 50%, hence all the plant extracts investigated were considered non-toxic. The relatively low toxicity of these plant extracts was also reported using African monkey kidney (Vero cells), mouse breast cancer cells (4T1) and liver carcinoma cell line (Hep2 cells) cells. Further, in the brine shrimp assay, all dichloromethane extracts of the studied parts of the plant species demonstrated minimal to no toxicity levels. These results also provided credence to the medicinal usage of these plants [46]. 

However, further studies are still needed to elucidate the environmental toxicity of the important insecticidal chemicals from these genera for their future application in the field. 

## 6. Future Outlook

The comparative safety of botanical insecticides from Meliaceae for humans, animals, the environment, and entomophagous predators has created a good opportunity for the development and utilization of these plant-derived pesticidal molecules. 

The systemic azadirachtin is a world-recognized and excellent botanical insecticide [157,158]. However, there are some chemicals with excellent activities from these fifteen genera, as they were shown in Table 2, Table 3 and Table 4. For examples:

The insecticidal activities of khayasin from *Neobeguea* against the coconut leaf beetle were similar to those of rotenone. Therefore, it was a promising candidate insecticide for the control of the coconut leaf beetle. Moreover, beddomei lactone and 3β,24,25-trihydroxycycloartane could be used as an active principle during the preparation of botanical insecticides for insect pest; like rice leaf-folder and mosquitoes. Moreover, the insecticidal activity of trisinlin A against newly hatched larvae of *S. litura* was comparable to azadirachtin, while the insecticidal activity of humilinolides A–E and methyl-2-hydroxy-3β-isobutyroxy-1-oxomeliac-8(30)-enate on the European corn borer was comparable to toosendanin.

It was noteworthy that chemicals from *Turraea* showed excellent insecticidal activity. In particular, larvicidal activities of 12α-diacetoxywalsuranolide, 11β,12α-diacetoxycedrelone, 1α,7α,12α-triacetoxy-4α-carbomethoxy-11β-hydroxy-14β,15β-epoxyhavanensin, and 11-epi-21-hydroxytoonacilide on the tomato leaf miner were more active than that of azadirachtin, while larvicidal activity of 1α,7α,11β-triacetoxy-4α-carbomethoxy-12α-(2-methylpropanoyloxy)-14β,15β-epoxyhavanensin and 1α,11β-diacetoxy-4α-carbomethoxy-7α-hydroxy-12α-(2-methylpropanoyloxy)-15-oxohavanensin, and 1α-acetoxy-3α-propanoyloxy-vilasinin on the malaria mosquito were more potent than that of azadirachtin. Among them 1α,7α,11β-triacetoxy-4α-carbomethoxy-12α-(2-methylpropanoyloxy)-14β,15β-epoxyhavanensin was active both on the tomato leaf miner and the mosquito. Additionally, the antifeedant activity of nilotin against the 4th instar Colorado potato beetle was comparable to that of limonin. 

Overall, the above-mentioned triterpenoids may be good candidates as lead compounds in the development of new insecticides for pest management. Still, other active compounds, such as nymania-3, trichilin, azadirone, and prieurianin obtainable from these 15 genera of the family Meliaceae, are still in the infant stage of their research and development. These chemicals also have good activity and deserve further studies. The activity of these compounds against insects should be systematically evaluated, and their effects on non-target organisms and the environment should be further evaluated. Moreover, the mechanisms of action, structure–activity relationship, and biosynthetic pathways of these chemicals are also worthy of further research. 

Still, there are some other factors hindering the practical use of the botanical active ingredients, including a short period of persistence of effectiveness, lack of plant material, insufficient information on effectiveness on target and non-target organisms, etc. Therefore, for the really highly effective chemicals, such as khayasin and beddomei lactone, research on the persistence and degradation, biosynthesis and the environmental toxicity should be systematically carried out in the future.

## Figures and Tables

**Figure 1:**
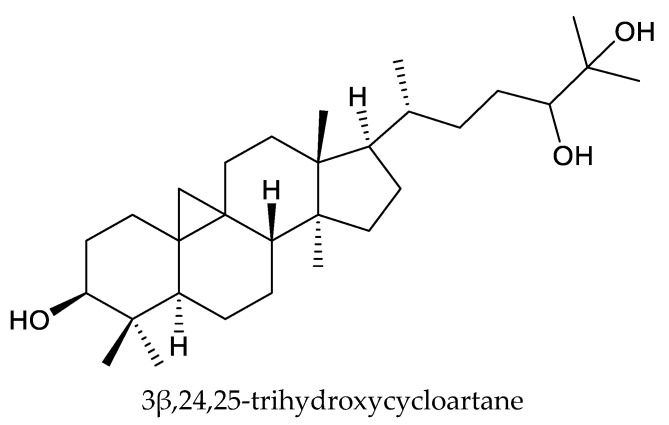
Structures of cycloartane chemicals.

**Figure 2:**
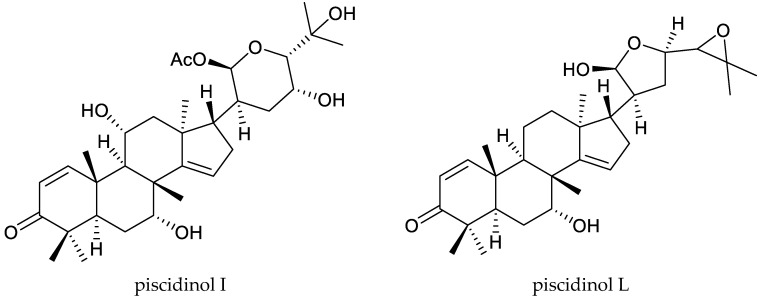
Structures of apotirucallane-type terpenoids.

**Figure 3:**
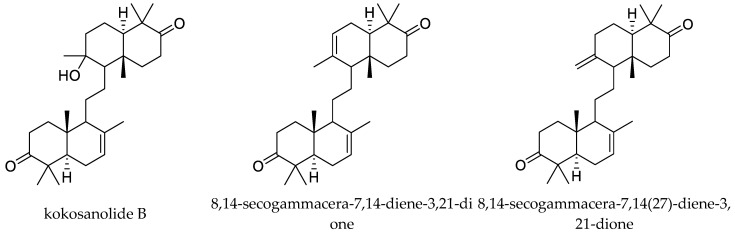
Structures of onoceranoid-type triterpenoids.

**Figure 4:**
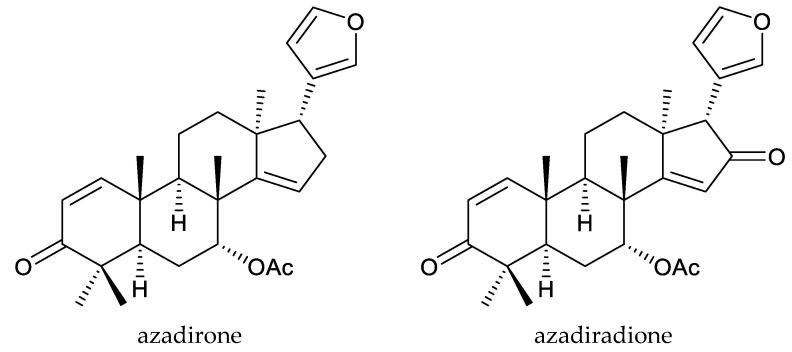
Structures of ring intact limonoids: azadirone-class chemicals.

**Figure 5:**
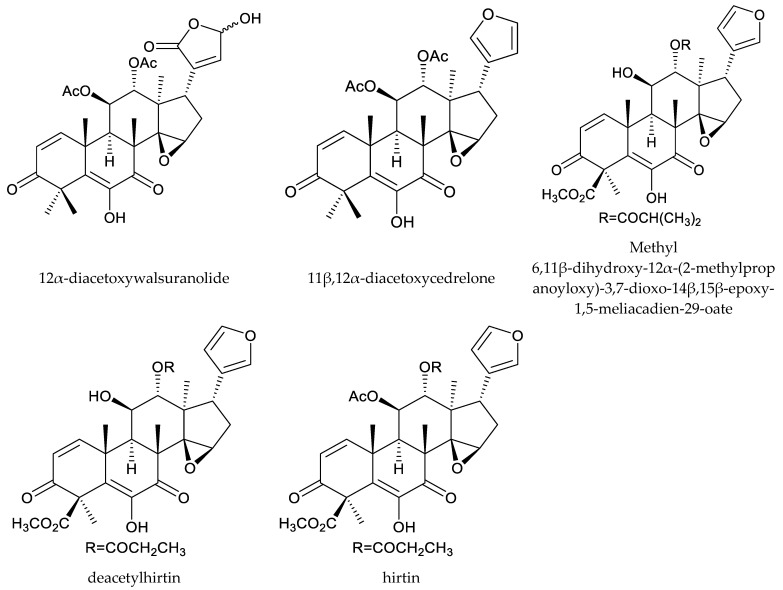
Structures of ring intact limonoids: cedrelone-class chemicals.

**Figure 6:**
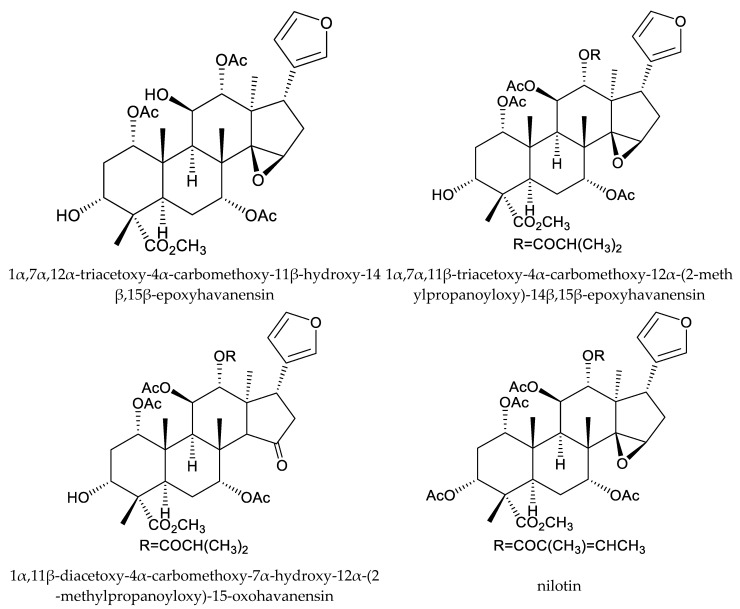

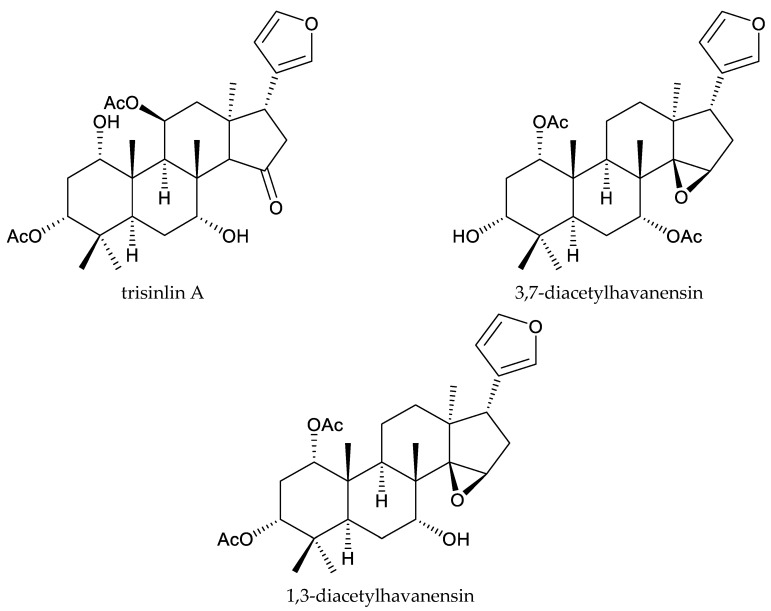
Structures of ring intact limonoids: havanensin-class chemicals.

**Figure 7:**
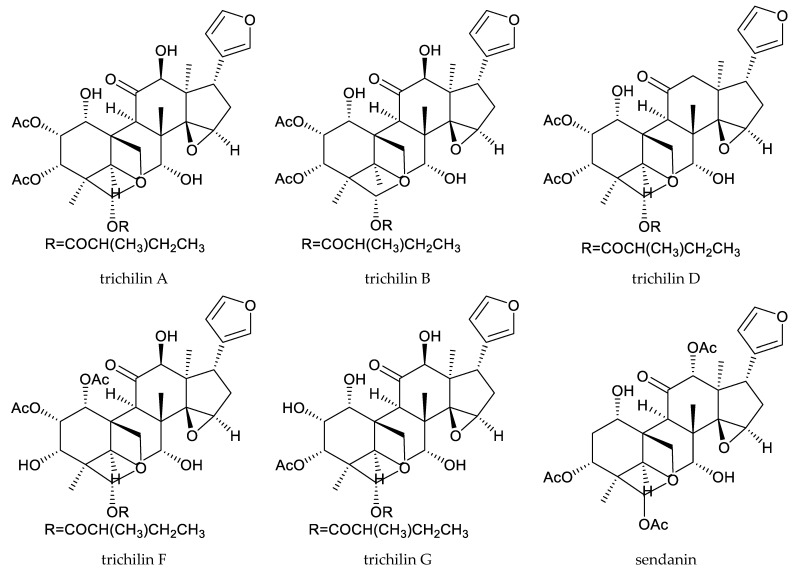
Structures of ring intact limonoids: trichilin-class chemicals.

**Figure 8:**
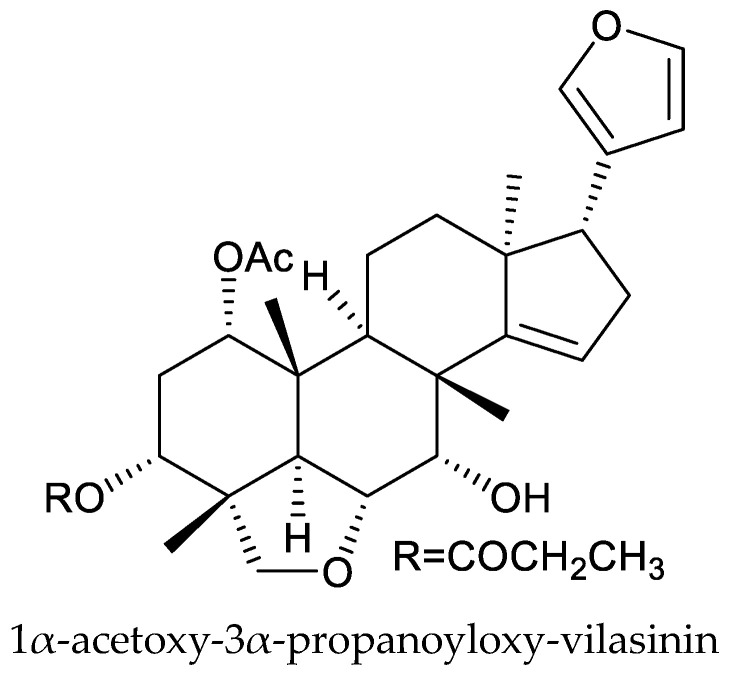
Structures of ring intact limonoids: vilasinin-class chemicals.

**Figure 9:**
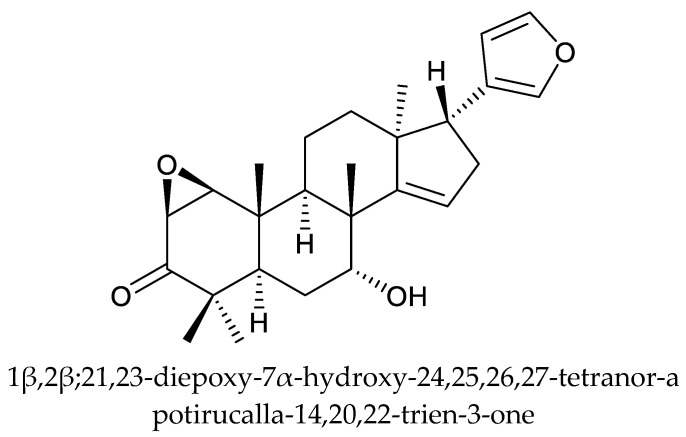
Structures of ring intact limonoids: other chemicals.

**Figure 10:**
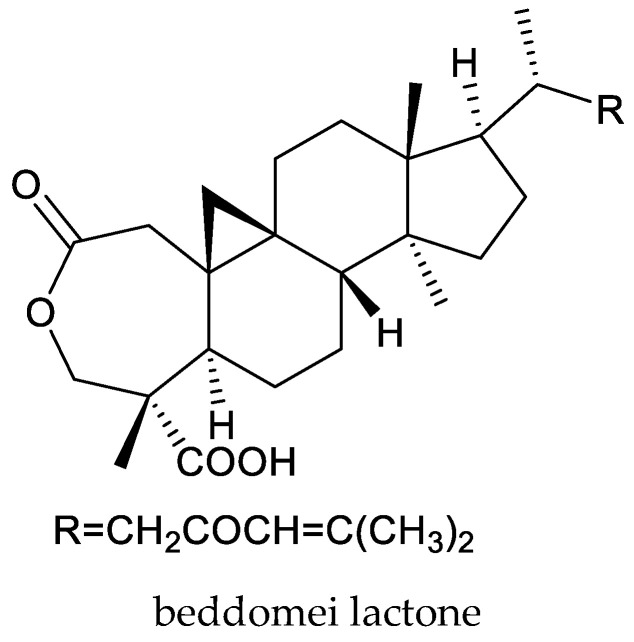
Structures of ring A-seco group chemicals.

**Figure 11:**
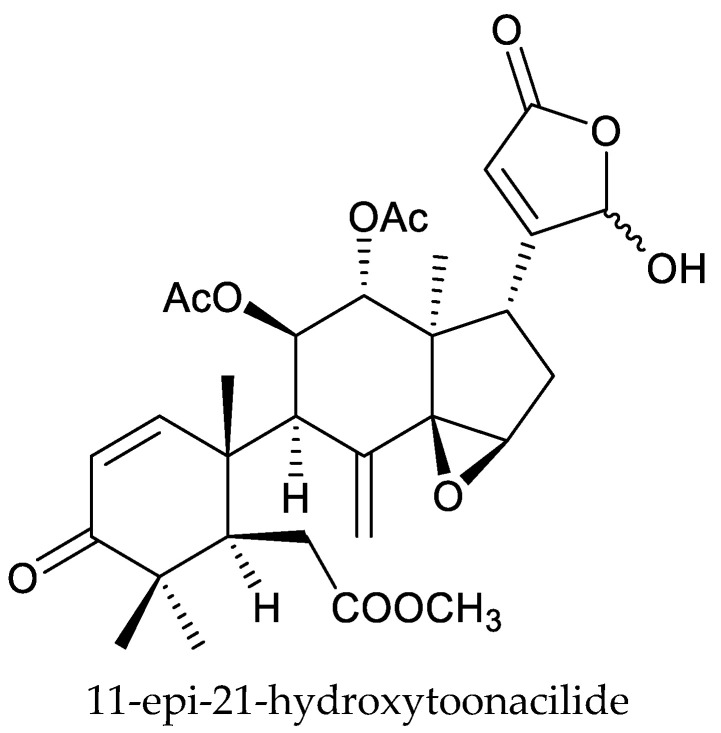
Structures of ring B-seco group chemicals.

**Figure 12:**
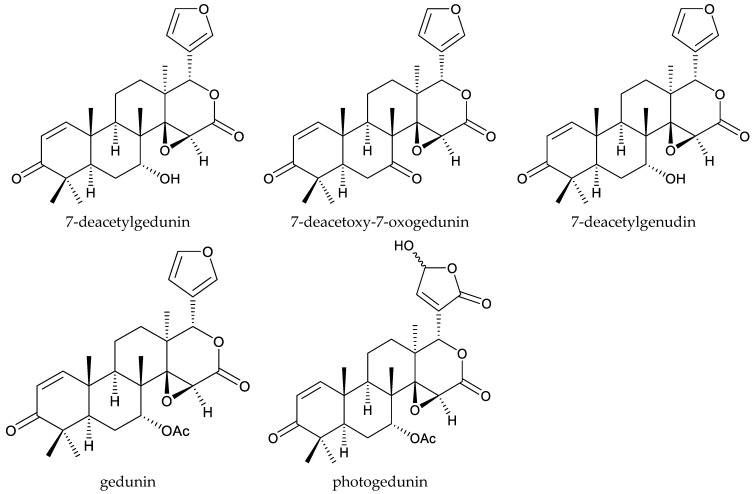
Structures of ring D-seco group chemicals.

**Figure 13:**
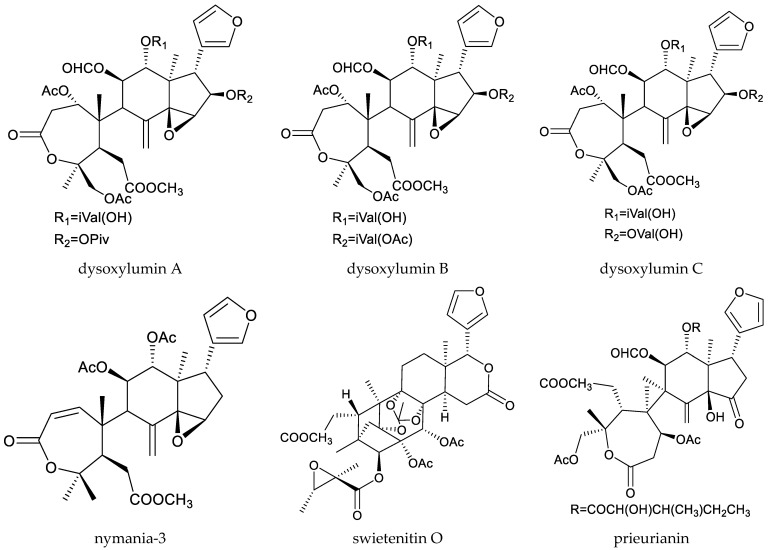
Structures of rings A,B-seco group: prieurianin-class chemicals.

**Figure 14:**
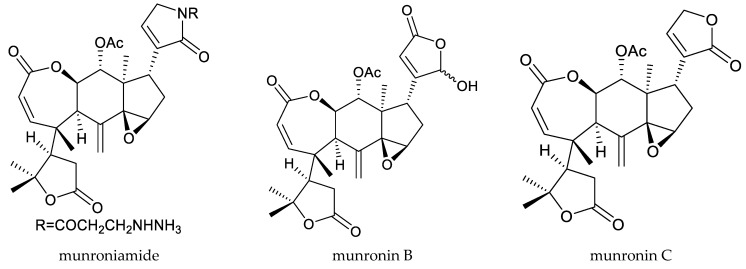

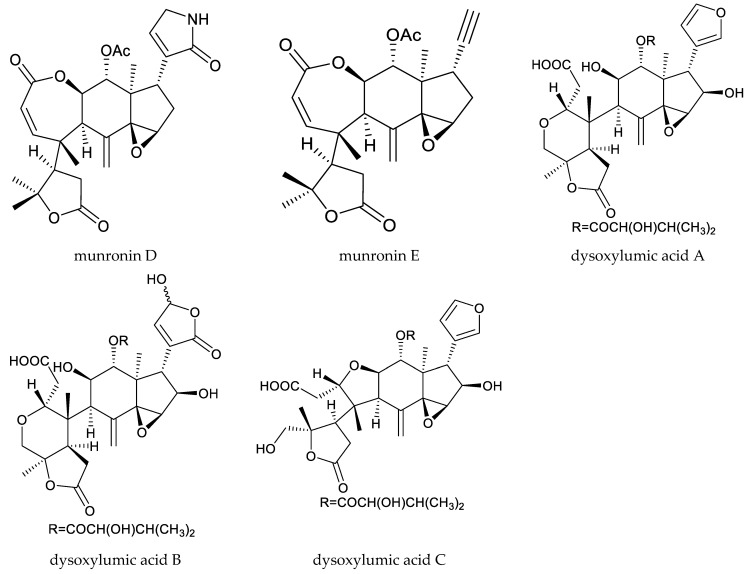
Structures of rings A,B-seco group: other chemicals.

**Figure 15:**
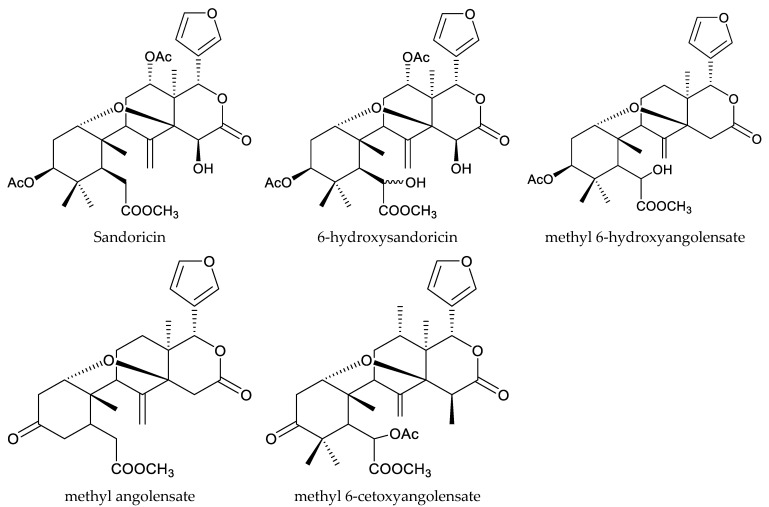
Structures of rings B,D-seco group: andirobin-class chemicals.

**Figure 16:**
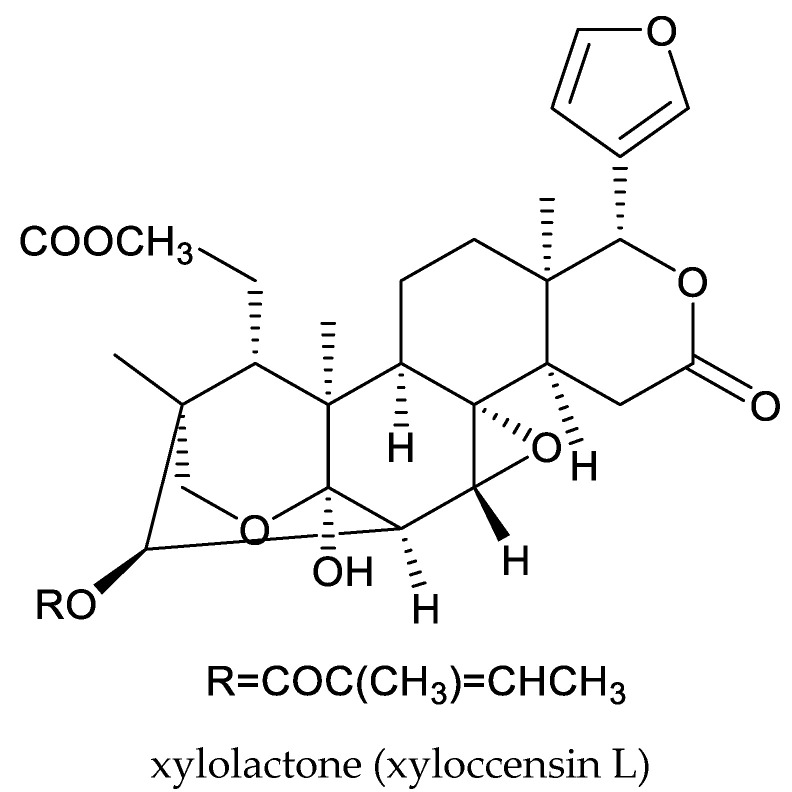
Structures of rings A,B,D-seco group chemicals.

**Figure 17:**
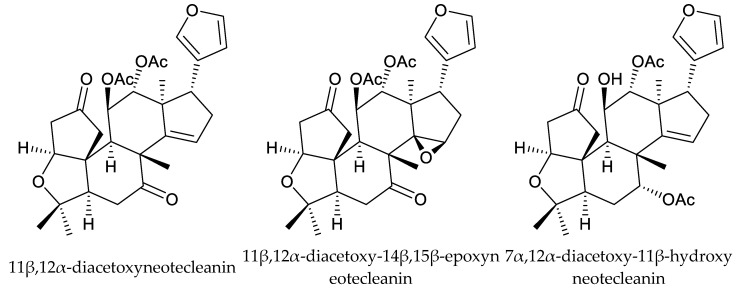
Structures of 1,n-linkage group chemicals.

**Figure 18:**
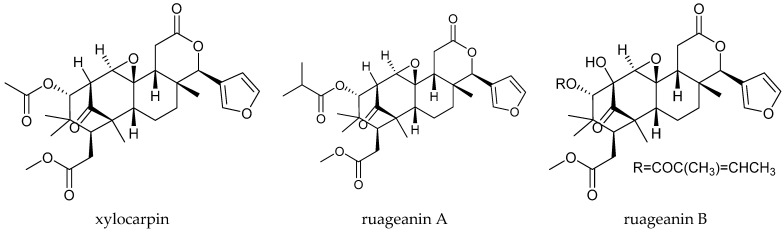

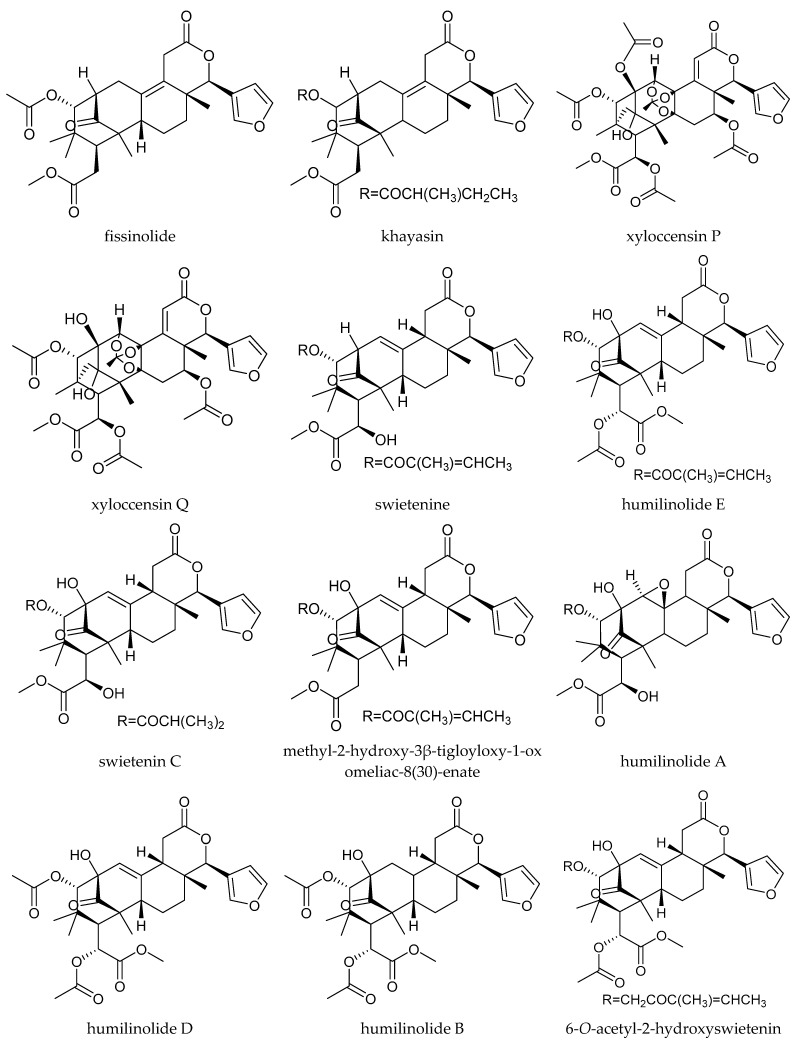

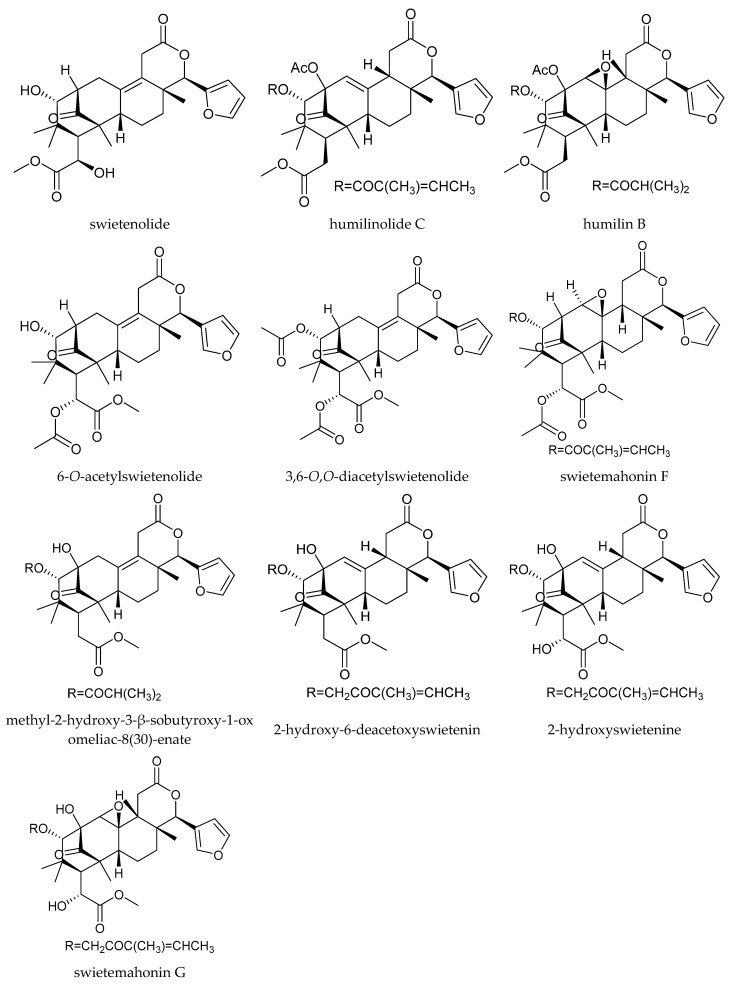
Structures of 2,30-linkage group: mexicanolide-class chemicals.

**Figure 19:**
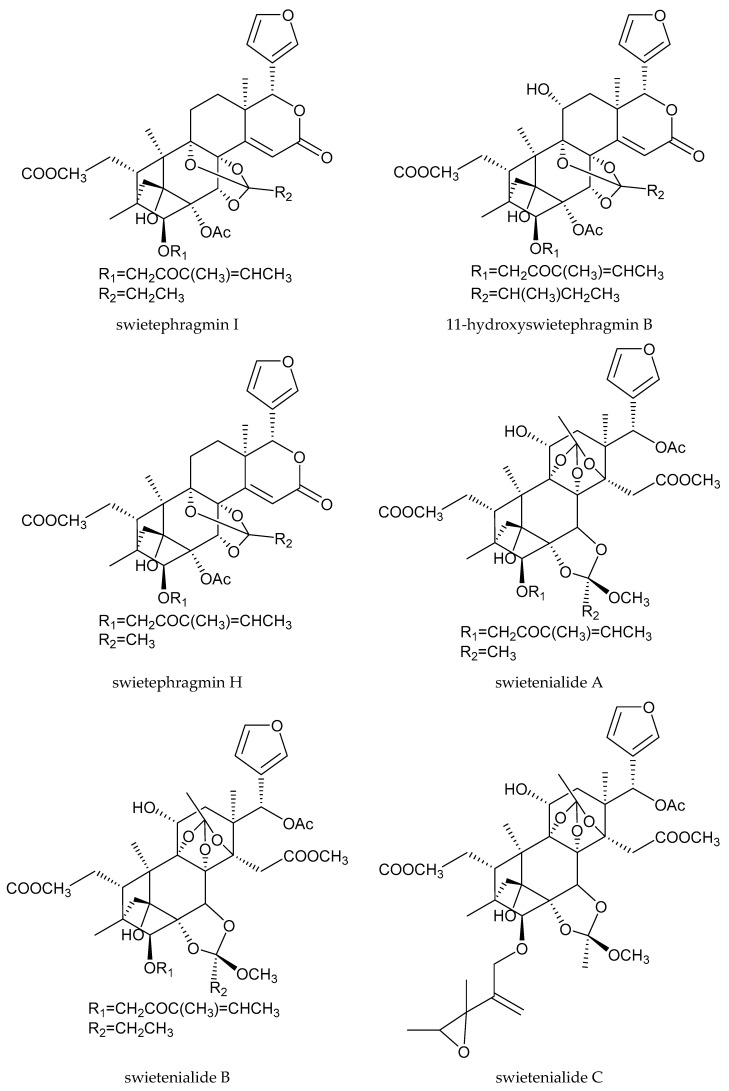

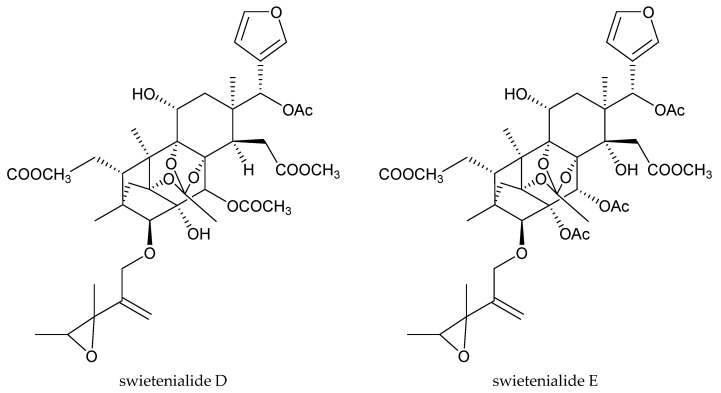
Structures of 2,30-linkage group: phragmalin-class chemicals.

**Figure 20:**
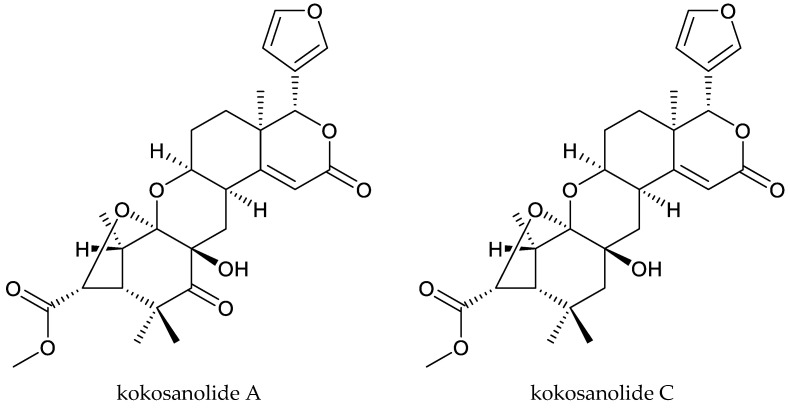
Structures of kokosanolides-type tetranortriterpenoids.

**Table 1:** The 37 insecticidal plant species of 15 genera (*Munronia*, *Neobeguea*, *Pseudocedrela*, *Nymania*, *Quivisia*, *Ruagea*, *Dysoxylum*, *Soymida*, *Lansium*, *Sandoricum*, *Walsura*, *Trichilia*, *Swietenia*, *Turraea* and *Xylocarpus*) in Meliaceae.

Family	Genus	Species
Meliaceae	*Munronia*	*Munronia henryi* Harms
	*Neobeguea*	*Neobeguea mahafalensis* J.-F. Leroy
	*Pseudocedrela*	*Pseudocedrela kotschyi* (Schweinf.) Harms
	*Nymania*	*Nymania capensis* (Thunb.) Lindb.
	*Quivisia*	*Quivisia papinae* Baillon ex Grandidier
	*Ruagea*	*Ruagea glabra* Triana and Planch.
	*Dysoxylum*	*Dysoxylum beddomei* Hiern
		*Dysoxylum malabaricum* Bedd.
		*Dysoxylum hainanense* (Merr.)
	*Soymida*	*Soymida febrifuga* (Roxb.) A. Juss.
	*Lansium*	*Lansium domesticum* Corr.
	*Sandoricum*	*Sandoricum koetjape* (Burm.f.) Merr.
	*Walsura*	*Walsura trifoliata* (A. Juss.) Harms. (synonym: *Walsura piscidia* Roxb.)
	*Trichilia*	*Trichilia elegans* A. Juss.
		*Trichilia catigua* A. Juss.
		*Trichilia roka* Chiov.
		*Trichilia havanensis* Jacq.
		*Trichilia sinensis* Bentv.
		*Trichilia hirta* L.
		*Trichilia pallida* Swartz
		*Trichilia claussenii* Catiguá
		*Trichilia pallens* C. DC.
		*Trichilia emetica* Vahl.
		*Trichilia gilgiana* Harms
		*Trichilia americana* Sessé and Moc.
	*Swietenia*	*Swietenia humilis* Zucc.
		*Swietenia macrophylla* King
		*Swietenia mahogani* JACQ.
	*Turraea*	*Turraea obtusifolia* Hochstetter
		*Turraea abyssinica* Hochst.
		*Turraea floribunda* Hochstetter
		*Turraea wakefieldii* Oliv.
		*Turraea nilotica* Kotschy and Peyr.
		*Turraea parvifolia* Defl.
	*Xylocarpus*	*Xylocarpus granatum* J. Koenig
		*Xylocarpus moluccensis* (Lam.) M. Roem.
		*Xylocarpus obovatus* (Blume) A. Juss.

**Table 2:** Antifeedant activity of insecticidal triterpenoids of plants from 15 genera in Meliaceae.

Compound	Plant Source	Insect	Activity	Refs.
fissinolide	*Soymida febrifuga*	*Achaea janata*	AI = 76.46 μg/cm^2^	[2]
		*Spodoptera litura*	AI = 61.69 μg/cm^2^	
swietenitin O	*Soymida febrifuga*	*Achaea janata*	AI = 66.61 μg/cm^2^	[2]
		*Spodoptera litura*	AI = 51.93 μg/cm^2^	
xylolactone (xyloccensin L)	*Xylocarpus granatum*	*Piece brassicae*	AFD at 1000 μg/mL	[3]
methyl 6-acetoxyangolensate	*Lansium domesticum*	*Spodoptera littoralis*	AFD at 500 μg/mL	[11,89]
munroniamide	*Munronia henryi*	*Pieris brassicae* L.	AR = 27.6% at 1000 μg/mL (48 h)	[19]
munronins B	*Munronia henryi*	*Pieris brassicae* L.	AR = 20.9% at 1000 μg/mL (48 h)	[20]
munronins C	*Munronia henryi*	*Pieris brassicae* L.	AR = 31.0% at 1000 μg/mL (48 h)	[20]
munronins D	*Munronia henryi*	*Pieris brassicae* L.	AR = 28.0% at 1000 μg/mL (48 h)	[20]
munronins E	*Munronia henryi*	*Pieris brassicae* L.	AR = 37.1% at 1000 μg/mL (48 h)	[20]
methyl angolensate	*Neobeguea mahafalensis* *Ruagea glabra* *Lansium domesticum* *Trichilia elegans* *Xylocarpus granatum* *Xylocarpus moluccensis*	*Spodoptera litura*	PFI = 65.3	[26]
nymania-3	*Nymania capensis* *Dysoxylum malabaricum*	*Pericallia ricini*	AFD at 1–10 μg/cm^2^ leaf	[50]
melianone	*Quivisia papinae* *Guarea grandiflora*	*Reticulitermes speratus*	95% mortality, 30 d, at 100 μg/disc	[55]
			AFD at 100 μg/disc	
xylocarpin	*Ruagea glabra*	*Spodoptera frugiperda*	AI = 77.8 at 1000 μg/mL (18 h)	[58]
ruageanin A	*Ruagea glabra*	*Spodoptera frugiperda*	AI = 72.6 at 1000 μg/mL (18 h)	[58]
ruageanin B	*Ruagea glabra*	*Spodoptera frugiperda*	AI = 86.3 at 1000 μg/mL (18 h)	[58]
dysoxylumic acid A	*Dysoxylum hainanense*	*Pieris rapae* L.	AR = 78.7% at 500 μg/mL	[65]
dysoxylumic acid B	*Dysoxylum hainanense*	*Pieris rapae* L.	AR = 64.1% at 500 μg/mL	[65]
dysoxylumic acid C	*Dysoxylum hainanense*	*Pieris rapae* L.	AR = 59.4% at 500 μg/mL	[65]
dysoxylumin A	*Dysoxylum hainanense*	*Pieris rapae* L.	AR = 73.8% at 1000 μg/mL	[65]
dysoxylumin B	*Dysoxylum hainanense*	*Pieris rapae* L.	AR = 77.4% at 1000 μg/mL	[65]
dysoxylumin C	*Dysoxylum hainanense*	*Pieris rapae* L.	AR = 74.9% at 1000 μg/mL	[65]
kokosanolide A	*Lansium domesticum*	*Epilachna vigintioctopunctata*	AF = 78%	[82]
kokosanolide B	*Lansium domesticum*	*Epilachna vigintioctopunctata*	AF = 99%	[82]
8,14-secogammacera-7,14-diene-3,21-dione	*Lansium domesticum*	*Epilachna vigintioctopunctata*	AF = 85%	[82]
8,14-secogammacera-7,14(27)-diene-3,21-dione	*Lansium domesticum*	*Epilachna vigintioctopunctata*	AF = 56%	[82]
swietemahonin G	*Swietenia mahogani*	*Spodoptera littoralis*	AFD at 300 μg/mL	[88]
swietephragmin I	*Swietenia mahogani*	*Spodoptera littoralis*	AFD at 500 μg/mL	[88]
2-Hydroxy-6-deacetoxyswietenin	*Swietenia mahogani*	*Spodoptera littoralis*	AFD at 500 μg/mL	[88]
6-O-acetyl-2-hydroxyswietenin	*Swietenia mahogani*	*Spodoptera littoralis*	AFD at 500 μg/mL	[88]
2-hydroxyswietenine	*Swietenia mahogani*	*Spodoptera littoralis*	AFD at 500 μg/mL	[88]
methyl 6-hydroxyangolensate	*Swietenia mahogani* *Lansium domesticum*	*Spodoptera littoralis*	AFD at 500 μg/mL	[88]
7-deacetoxy-7-oxogedunin	*Swietenia mahogani* *Swietenia macrophylla*	*Spodoptera littoralis*	AFD at 1000 μg/mL	[88]
swietephragmin H	*Swietenia mahogani*	*Spodoptera littoralis*	AFD at 1000 μg/mL	[88]
11-hydroxy-swietephragmin B	*Swietenia mahogani*	*Spodoptera littoralis*	AFD at 1000 μg/mL	[88]
sandoricin	*Sandoricum koetjape*	*Ostrina nubilalis*	AFD at 200 μg/mL	[92]
		*Spodoptera frugiperda*	AFD at 25 μg/mL	
6-hydroxysandoricin	*Sandoricum koetjape*	*Ostrina nubilalis*	AFD at 200 μg/mL	[92]
		*Spodoptera frugiperda*	AFD at 25 μg/mL	
azadiradione	*Quivisia papinae* *Lansium domesticum*	*Plutella xylostella*	AR = 90.6% at 2000 μg/mL (48 h)	[96]
trichilin D	*Trichilia roka*	*Spodoptera eridania*	AFD at 400 μg/mL	[114]
trichilin F	*Trichilia roka*	*Spodoptera littoralis*	AFD at 300 μg/mL	[114]
trichilin G	*Trichilia roka*	*Spodoptera littoralis*	AFD at 300 μg/mL	[114]
trichilin B	*Trichilia roka*	*Spodoptera exigua*	MIC = 200 μg/mL	[116]
azadirone	*Trichilia havanensis*	*Leptinotarsa decemlineata*	AI = 11.6–26.9, at 100–500 μg/mL	[119]
1β,2β;21,23-diepoxy-7α-hydroxy-24,25,26,27-tetranor-apotirucalla- 14,20,22-trien-3-one	*Trichilia havanensis*	*Leptinotarsa decemlineata*	AFD at 300 μg/mL	[119]
Methyl 6,11β-dihydroxy-12α-(2-methylpropanoyloxy)-3,7-dioxo-14β,15β-epoxy-1,5-meliacadien-29-oate	*Trichilia pallida*	*Heliothis virescens*	FI = 29	[120]
		*Helicoverpa armigera*	FI = 32	
deacetylhirtin	*Trichilia pallida*	*Heliothis virescens*	FI = 49	[120]
		*Helicoverpa armigera*	FI = 42	
swietenialide A	*Swietenia mahogani*	*Spodoptera littoralis*	AFD at 1000 μg/mL	[130]
swietenialide B	*Swietenia mahogani*	*Spodoptera littoralis*	AFD at 1000 μg/mL	[130]
swietenialide C	*Swietenia mahogani*	*Spodoptera littoralis*	AFD at 1000 μg/mL	[130]
swietenialide D	*Swietenia mahogani*	*Spodoptera littoralis*	AFD at 1000 μg/mL	[130]
swietenialide E	*Swietenia mahogani*	*Spodoptera littoralis*	AFD at 1000 μg/mL	[130]
swietenine	*Swietenia macrophylla* *Swietenia mahagoni*	*Spodoptera frugiperda*	DC_50_ = 0.19 mg L^−1^	[131]
swietenolide	*Swietenia mahogani*	*Spodoptera frugiperda*	AI = 94.1, at 1000 μg/mL	[132]
6-*O*-acetylswietenolide	*Swietenia mahogani*	*Spodoptera frugiperda*	AI = 72.2, at 1000 μg/mL	[132]
3,6-*O,O*- diacetylswietenolide	*Swietenia mahogani*	*Spodoptera frugiperda*	AI = 72.0, at 1000 μg/mL	[132]
swietemahonin F	*Swietenia mahogani*	*Spodoptera frugiperda*	AI = 70.2, at 1000 μg/mL	[132]
Nilotin	*Turraea nilotica*	*Leptinotarsa decemlineata*	ED_50_ = 7 μg/mL	[137]
7-deacetylgenudin	*Xylocarpus granatum Pseudocedrela kotschyi*	*Reticulitermes speratus*	PC_95_ = 113.7 μg/disc	[144]
xyloccensin P	*Xylocarpus granatum*	*Mythimna separata*	AFD at 500 μg/mL	[149]
xyloccensin Q	*Xylocarpus granatum*	*Mythimna separata*	AFD at 500 μg/mL	[149]

**Table 3:** Poisonous activity of insecticidal triterpenoids of plants from 15 genera in Meliaceae.

Compound	Plant Source	Insect	Activity	Ref.
swietenitin O	*Soymida febrifuga*	*Achaea janata*	LC_50_ = 0.65 μg/cm^2^	[2]
		*Spodoptera litura*	LC_50_ = 0.75 μg/cm^2^	
methyl angolensate	*Neobeguea mahafalensis* *Ruagea glabra* *Lansium domesticum* *Trichilia elegans* *Xylocarpus granatum* *Xylocarpus moluccensis*	*Spodoptera frugiperda*	mortality rate of 40% at 50 mg·kg^−1^	[27]
khayasin	*Xylocarpus moluccensis*	*Brontispa longissima*	LC_50_ = 7.28 μg/mL (24 h)	[28]
photogedunin	*Cedrela fissilis* *Xylocarpus granatum*	*Atta sexdens rubropilosa*	S_50_ = 9 days	[44]
7-deacetoxy-7-oxogedunin	*Pseudocedrela kotschyi*	*Atta sexdens rubropilosa*	S_50_ = 11 days	[44]
7-deacetylgedunin	*Pseudocedrela kotschyi*	*Atta sexdens rubropilosa*	S_50_ = 9 days	[44]
piscidinol I	*Walsura trifoliata*	*Achaea janata*	LC_50_ = 40.83 mg/cm^2^	[96]
		*Spodoptera litura*	LC_50_ = 46.55 mg/cm^2^	
piscidinol L	*Walsura trifoliata*	*Achaea janata*	LC_50_ = 20.00 mg/cm^2^	
		*Spodoptera litura*	LC_50_ = 22.02 mg/cm^2^	
trisinlin A	*Trichilia sinensis*	*Spodoptera litura*	96.67% mortalities, 14 d, at 20 μg/mL	[111]
trichilin A	*Trichilia emetica* *Trichilia roka*	*Spodoptera eridania*	killed the third instar larvae over a 10-day feeding	[114]
humilinolide A	*Swietenia humilis*	*Ostrinia nubilalis*	larval mortality: 43.3% at 50 μg/mL	[123]
humilinolide B	*Swietenia humilis*	*Ostrinia nubilalis*	larval mortality: 50% at 50 μg/mL	[123]
humilinolide C	*Swietenia humilis*	*Ostrinia nubilalis*	larval mortality: 50% at 50 μg/mL	[123]
humilinolide D	*Swietenia humilis*	*Ostrinia nubilalis*	larval mortality: 63.3% at 50 μg/mL	[123]
humilinolide E	*Swietenia humilis*	*Ostrinia nubilalis*	the survival rate: 20%	[133]
humilin B	*Swietenia humilis*	*Ostrinia nubilalis*	the survival rate > 50%	[133]
swietenin C	*Swietenia humilis*	*Ostrinia nubilalis*	the survival rate < 50%	[133]
methyl-2-hydroxy-3-β-isobutyroxy-1 -oxomeliac-8(30)-enate	*Swietenia humilis*	*Ostrinia nubilalis*	the survival rate: 30%	[133]
methyl-2-hydroxy-3β-tigloyloxy-1-oxomeliac-8(30)-enate	*Swietenia humilis*	*Ostrinia nubilalis*	the survival rate > 60%	[133]
12α-diacetoxywalsuranolide	*Turraea abyssinica*	*Tuta absoluta*	LD_50_ = 6.6 μg/mL	[135]
1α,7α,12α-triacetoxy-4α-carbomethoxy-11β-hydroxy- 14β,15β-epoxyhavanensin	*Turraea abyssinica*	*Tuta absoluta*	LD_50_ = 4.6 μg/mL	[135]
11-epi-21-hydroxytoonacilide	*Turraea abyssinica*	*Tuta absoluta*	LD_50_ = 7.1 μg/mL	[135]
11β,12α-diacetoxycedrelone	*Turraea abyssinica*	*Tuta absoluta*	LD_50_ = 5.8 μg/mL	[135]
1α,7α,11β-triacetoxy-4α-carbomethoxy-12α -(2-methylpropanoyloxy)-14β,15β-epoxyhavanensin	*Turraea floribunda*	*Anopheles gambiae*	LD_50_ = 4.0 μg/mL	[136]
1α,11β-diacetoxy-4α-carbomethoxy -7α-hydroxy-12α-(2-methylpropanoyloxy)-15- oxohavanensin	*Turraea floribunda*	*Anopheles gambiae*	LD_50_ = 3.6 μg/mL	[136]
1α-acetoxy-3α- propanoyloxy-vilasinin	*Turraea wakefieldii* *Turraea parvifolia*	*Anopheles gambiae*	LD_50_ = 7.1 μg/mL	[136]
11β,12α-diacetoxyneotecleanin	*Turraea wakefieldii*	*Anopheles gambiae*	LD_50_ = 7.83 μg/mL (24 h)	[138]
11β,12α-diacetoxy-14β,15β-epoxyneotecleanin	*Turraea wakefieldii*	*Anopheles gambiae*	LD_50_ = 7.07 μg/mL (24 h)	[138]
11β,12α-diacetoxy-11β-hydroxyneotecleanin Anophelesgambiae	*Turraea wakefieldii*	*Anopheles gambiae*	LD_50_ = 7.05 μg/mL (24 h)	[138]
gedunin	*Xylocarpus granatum* *Xylocarpus obovatus*	*Spodoptera frugiperda*	LC_50_ = 39.0 μg/mL	[148]

**Table 4:** Growth regulatory activity of insecticidal triterpenoids of plants from 15 genera in Meliaceae.

Compound	Plant Source	Insect	Activity	Ref.
prieurianin	*Nymania capensis* *Trichilia firieuriana* *Turraea obtusifolia*	*Helicoverpa armigera*	EC_50_ = 18.8 μg/mL	[49]
azadiradione	*Quivisia papinae* *Lansium domesticum*	*Heliothis uirescens*	EC_50_ = 560 μg/mL	[53]
3β,24,25-trihydroxycycloartane	*Dysoxylum beddomei*	*Cnaphalocrocis medinalis*	prolonged larval duration and reduced larval weight at 3–12 μg/mL	[68]
beddomei lactone	*Dysoxylum beddomei*	*Cnaphalocrocis medinalis*	prolonged larval duration and reduced larval weight at 3–12 μg/mL	[68]
sendanin	*Trichilia roka*	*Pectinophora gossypiella Heliothis zea* *Heliothis virescens* *Spodoptera frugiperda*	ED_50_ = 9 μg/mL ED_50_ = 55 μg/mL ED_50_ = 60 μg/mL ED_50_ = 11 μg/mL	[117]
azadirone	*Trichilia havanensis*	*Leptinotarsa decemlineata*	AI valus at 100-500 μg/mL varied from 11.6 to 26.9	[119]
hirtin	*Trichilia hirta* *Trichilia pallida*	*Peridroma saucia*	EC_50_ = 13.0 μg/mL (7 d)	[121]
humilinolide C	*Swietenia humilis*	*Ostrinia nubilalis*	decreased the growth, 5 μg/mL	[123]
			13% pupation, 50 μg/mL	[123]
humilinolide D	*Swietenia humilis*	*Ostrinia nubilalis*	10% pupation, 50 μg/mL	[123]
